# Prophylactic Donor Lymphocyte Infusions in Pediatric Patients With High‐Risk Hematological Malignancies

**DOI:** 10.1111/ejh.70149

**Published:** 2026-03-05

**Authors:** Denise Epple, Katja Gall, Luisa Paschke, Hans‐Jochem Kolb, Angela Wawer, Hans Knabe, Irene von Luettichau, Julia Hauer, Uwe Thiel

**Affiliations:** ^1^ Department of Pediatrics, Children's Cancer Research Center, Kinderklinik München Schwabing, TUM School of Medicine Technical University of Munich Munich Germany; ^2^ Department of Hematology‐Oncology Immunology Infectious Diseases Klinikum München Schwabing Munich Germany; ^3^ Bavarian Stem Cell Bank (Bayerische Stammzellbank) Gauting Germany

**Keywords:** donor lymphocyte infusion, leukemia, pediatrics, stem cell transplantation

## Abstract

**Introduction:**

Allogeneic stem cell transplantation (allo‐SCT) and donor lymphocyte infusions (DLI) can elicit a graft‐versus‐leukemia (GvL) effect in pediatric patients with hematological malignancies. We report our single‐center experience with prophylactic DLI in high‐risk pediatric patients with leukemia or lymphoma, focusing on feasibility, safety, and efficacy.

**Methods:**

In total, 10 high‐risk patients received prophylactic DLI following allo‐SCT. Donors were either matched (*n* = 5) or haploidentical (*n* = 5). CD3+ T‐cell doses of up to 1 × 10^7^ cells/kg body weight were administered, in some cases over extended periods exceeding three years.

**Results:**

The treatment was associated with a favorable toxicity profile. In our cohort, 40% of patients developed moderate acute (*n* = 2) or chronic (*n* = 2) graft‐versus‐host disease (GvHD); no cases of severe or high grade GvHD occurred. Given the high‐risk profile of our cohort, outcomes were encouraging, with relapse‐free survival (RFS) of 70% and overall survival (OS) of 80% at a median follow‐up of 20.5 months. Especially, the two subgroups of patients with acute myeloid leukemia (AML) after relapse and patients who were transplanted in first complete remission (CR1) showed outcomes superior to currently reported data. One AML patient, who had experienced three relapses, received prophylactic DLI after a third allo‐SCT and remains in complete remission (CR), more than 3 years after the last allo‐SCT.

**Conclusion:**

These data suggest that prophylactic DLI may represent a safe and effective treatment option for pediatric patients with hematological malignancies at high risk of posttransplant relapse.

**Abbreviations**
ALCL: anaplastic large cell lymphoma
ALL: acute lymphoblastic leukemia
allo‐SCT: allogeneic stem cell transplantation
AML: acute myeloid leukemia
BOS: bronchiolitis obliterans syndrome
BSB: Bayerische Stammzellbank
CAR: chimeric antigen receptor
CML: chronic myeloid leukemia
CR1: first complete remission
DLI: donor lymphocyte infusions
GvHD: graft‐versus‐host disease
GvL: graft‐versus‐leukemia
MMFD: mismatched family donor
MRD: minimal residual disease
MSD: matched sibling donor
OS: overall survival
PBSC: peripheral blood stem cells
RFS: relapse‐free survival

## 1 Introduction

Hematological malignancies are the most common oncological diagnoses among pediatric patients, with leukemias accounting for approximately 32% and lymphomas accounting for approximately 12% of all cases globally [1]. As a result of clinical trials and multiagent chemotherapy, the outcome has improved notably over the past decades, with survival rates up to 81% across all cancer entities today [2]. However, patients with high‐risk features or relapse after therapy show a much poorer prognosis. For this group, myeloablative chemotherapy followed by allogeneic stem cell transplantation (allo‐SCT) is often the only curative option [3].

Recently, advances in immunotherapy have revolutionized the treatment of relapsed and refractory B‐cell lymphocytic leukemia and lymphoma patients. Several novel therapeutic options have become available, such as monoclonal antibody therapy and chimeric antigen receptor (CAR)‐T cell therapy targeting CD19 [4, 5]. However, other hematological malignancies, like myeloid neoplasms, are more complex to target, as they express similar surface antigens as hematopoietic stem cells and normal tissue. Several targets, like CD33 and CD123 for acute myeloid leukemia (AML) or CD30 for Hodgkin lymphoma and anaplastic large cell lymphoma (ALCL) [6, 7], have been addressed so far, but the results are not comparable to the success achieved with B‐cell neoplasms.

For those patients, another immunotherapeutic approach seems to be promising. Since the 1990s, it is assumed that the transplanted immune system has a potentially direct anti‐leukemia effect, for example, mediated by donor‐derived T‐cells that recognize tumor‐associated antigens [8, 9]. This is commonly referred to as the graft‐versus‐malignancy or graft‐versus‐leukemia (GvL) effect and has become one of the key mechanisms for the long‐term curative effect of allo‐SCT. Donor lymphocyte infusions (DLI) have consequently emerged as an important strategy to enhance GvL response in various hematological malignancies [10, 11].

Currently there are three different implementations in the use of DLI [12]. Prophylactic DLI can be given to high‐risk patients after allo‐SCT with no evidence of the underlying disease as a maintenance therapy in order to prevent disease recurrence. In case of incomplete donor chimerism, detectable minimal residual disease (MRD), or molecular or cytogenetic relapse, DLI can be administered pre‐emptively. The term therapeutic DLI describes the use of DLI in patients with overt hematological relapse.

However, the induction of graft‐versus‐host disease (GvHD) represents the most relevant complication of DLI and largely contributes to transplant‐related mortality, especially in elder patients. Multiple factors like graft source, cell dosage, application frequency, and adaptive use of immune‐suppressive medication influence the risk of post‐DLI GvHD. The incidence of acute GvHD Grade II–IV after DLI in adult patients is approximately 12%, and the incidence of chronic GvHD around 31% [13]. The delicate balance between GvL and GvHD remains the most significant challenge in the administration of DLI.

In adult patients, consensus‐based recommendations for the application of DLI after allo‐SCT have been defined based on retrospective studies and expert consensus [12, 13]. For pediatric patients, there is only limited experience, and up to date, general guidelines are missing. Nevertheless, many centers have already been applying DLI in pediatric cancer patients over the past years and have achieved positive results in individual cases [14]. But so far, no systematic analysis is available addressing important questions as the optimal treatment schedule, dosage, toxicities, or clinical efficacy unequivocally contributed to by DLI. Here we report the results of DLI in pediatric patients with a high risk of relapse after allo‐SCT.

## 2 Methods

### 2.1 Patients

In this retrospective study, we analyzed overall survival (OS), relapse‐free survival (RFS), and treatment‐related toxicity in posttransplantation patients who had received DLI between April 2019 and March 2025. All patients had undergone allo‐SCT for high‐risk hematological malignancy after remission induction treatment as recommended by the AIEOP‐BFM and NHL study groups. We included 8 patients with AML, one patient with chronic myeloid leukemia (CML) in blast phase, and one patient with ALCL, resulting in a total number of 10 patients. The study group consisted of seven male and three female patients; the median age at allo‐SCT was 10.6 years (range 4–17 years). Three patients received concomitant immunomodulatory medication during DLI administration in accordance with their genetic profile. The specific substances are reported in Table 2, the other patient characteristics are summarized in Table 1. Last follow‐up was March 31, 2025. As we describe retrospective data of a small heterogeneous group, we did not perform statistical significance tests.

**TABLE 1:** Patient characteristics.

Characteristic	All (*n* = 10; 100%)
Sex, *n* (%)	
Female	3 (30)
Male	7 (70)
Age at allo‐SCT (years)	
Median (range)	10.6 (4–17)
Disease, *n* (%)	
Acute myeloid leukemia	8 (80)
Chronic myeloid leukemia	1 (10)
Anaplastic large cell lymphoma	1 (10)
Remission at allo‐SCT, *n* (%)	
CR1	5 (50)
CR2	2 (20)
≥ CR3	3 (30)
Number of allo‐SCTs, *n* (%)	
1	6 (60)
2	3 (30)
3	1 (10)
Donor, *n* (%)	
MUD	5 (50)
MMFD	5 (50)
Stem cell source, *n* (%)	
BM	6 (60)
PBSCs	4 (40)

Abbreviations: Allo‐SCT, allogenic stem cell transplantation; BM, bone marrow; CR, complete remission; MMFD, haploidentical family donor; MUD, matches unrelated donor; PBSC, peripheral blood stem cells.

### 2.2 High‐Risk Criteria

The selected patients received allo‐SCT and subsequent DLI treatment for various high‐risk conditions. Relapse (*n* = 5) was the main indication for this intensified treatment (≥ CR2 at allo‐SCT). Three patients therefore had experienced two allo‐SCTs in their medical history and one patient had been transplanted for a third time. The remaining five patients fulfilled high‐risk criteria at initial diagnosis. Two patients had an unfavorable molecular profile (as described in Table 2), one patient presented with hyperleukocytosis and massive CNS infiltration, and another patient showed poor response to induction chemotherapy rendering refractory. The CML patient was transplanted due to blast phase at initial diagnosis.

**TABLE 2:** Individual characteristics and outcome.

	P#1	P#2	P#3		P#4	P#5	P#6	P#7	P#8	P#9	P#10
Sex	Female	Male	Male		Male	Male	Male	Male	Male	Female	Female
Age at most recent allo‐SCT (years)	4	17	5		9	6	16	5	15	14	15
Diagnosis	AML	AML	AML		AML	AML	ALCL	AML	AML	AML	CML
Indication for allo‐SCT	Third relapse (bm)	Molecular profile	Second relapse (bm)		Hyperleuko‐cytosis; CNS‐infiltration	First relapse	Second relapse	Molecular profile	First relapse (combined)	No CR after induction	CML blast phase
Molecular profile	t(7;12)(q36;p13)	FLT3‐ITD+	KMT2A‐MLLT3		KMT2A‐MLLT10	GATA1+ (mosaic DS)	ALK+	FLT3‐ITD+; mtRUNX1	FAB M1; MLL positive	KMT2A‐MLLT4	BCR‐ABL+
Date of allo‐SCT	Sep 2021	Nov 2022	Jan 2023		Jan 2023	Mar 2023	Jul 2023	Jul 2023	Aug 2023	Apr 2024	Aug 2024
Remission at allo‐SCT	CR4	CR1	CR3		CR1	CR2	CR3	CR1	CR2	CR1	CR1
Number of allo‐SCTs	3	1	2		1	1	2	1	2	1	1
Conditioning regimen	Fludarabin Treosulfan Thiotepa	Fludarabin Treosulfan Thiotepa	Fludarabin Amsacrin ARA‐C CYCLO Melphalan		Fludarabin Amsacrin ARA‐C CYCLO Melphalan	Busulfan Melphalan CYCLO	Etoposide TBI 8Gy	Busulfan Melphalan CYCLO	Busulfan Clofarabin Fludarabin	Fludarabin Treosulfan Thiotepa	Etoposide TBI 8Gy
Donor	MMFD	MUD	MMFD		MMFD	MUD	MMFD	MUD	MMFD	MUD	MUD
Stem cell source	BM	BM	BM		BM	BM	BM	PBSC	PBSC	PBSC	PBSC
GvHD prophylaxis	CsA/MMF/PT‐Cy	CsA/MTX	CsA/PT‐Cy		CsA/MMF/PT‐Cy	ATG/MTX/CsA	CsA/MMF/PT‐Cy	ATG/MTX/CsA	PT‐Cy/MMF/CsA	ATG/MTX/CsA	ATG/MTX/Tacrolimus
GvHD prior to DLI	No	No	Yes (aGvHD °II)		Yes (aGvHD °I)	No	Yes (aGvHD °I)	No	No	No	No
GvHD therapy	—	—	CsA/steroid/ruxolitinib		CsA/steroid/ruxolitinib	—	CsA/steroid	—	—	—	—
Time from alloSCT to DLI (months)	5	6	4		5	4	5	6	5	4	3
Number of DLI	21	6	9		12	3	1	8	5	2	4
Start dose DLI (CD3+ cells/kg bw)	5 × 10^4^	1 × 10^6^	1 × 10^5^		1 × 10^5^	1 × 10^5^	1 × 10^6^	1 × 10^5^	1 × 10^5^	1 × 10^5^	1 × 10^5^
Max. dose DLI (CD3+ cells/kg bw)	1 × 10^7^	1 × 10^7^	1 × 10^7^		1 × 10^7^	5 × 10^6^	—	1 × 10^7^	1 × 10^6^	5 × 10^5^	5 × 10^6^
Concomittant medication (target)	No	Sorafenib (FLT3‐ITD)	No		No	No	Alectinib (ALK)	Sorafenib (FLT3‐ITD)	No	No	Imatinib (BCR‐ABL)
GvHD after DLI	No	Yes	No		No	No	Yes	No	Yes	Yes	No
GvHD grade and localization	—	Mild chronic, mucosa	—		—	—	Acute °I, skin	—	Acute °I, skin	MODERATE chronic, lung	—
GvHD resolved	—	Yes	—		—	—	Yes	—	Yes	Yes	—
Relapse	No	No	Yes		No	Yes	No	No	Yes	No	No
Relapse free survival (months)	42	28	6		26	5	20	20	7	11	7
Follow‐up after allo‐SCT (months)	42	28	14		26	10	20	20	19	11	7
Death	No	No	Yes		No	Yes	No	No	No	No	No

Abbreviations: aGvHD, acute graft‐versus‐host disease; allo‐SCT, allogeneic stem cell transplantation; ARA‐C, cytarabine; ATG, antithymocyte globulin; bm, bone marrow; bw, body weight; CsA, cyclosporine A; CYCLO, cyclophosphamide; DLI, donor lymphocyte infusion; DS, down syndrome; GvHD, graft‐versus‐host disease; kg, kilogram; MMF, mycophenolatmofetil; MTX, methotrexate; PT‐Cy, posttransplant cyclophosphamide; TBI, total body irradiation.

### 2.3 Modalities of Administration

After allo‐SCT, all patients were monitored for hematopoietic chimerism and MRD in peripheral blood and/or bone marrow samples at given posttransplantation time points (approximately Days +30, +60, +90, and 6, 12, and 18 months). All patients and their legal guardians provided written informed consent prior to DLI. Patients were treated with DLI prophylactically starting from Day +100 after allo‐SCT due to high risk of relapse. In all patients, immunosuppressive therapy had been terminated at least 4 weeks prior to the first DLI and no signs of GvHD or infection were present.

The most frequently used starting T‐cell dose was 1 × 10^5^ CD3^+^ cells/kg body weight, according to previously published data [12, 15]. In P#1, we started with a slightly lower dose of 5 × 10^4^ CD3^+^ cells/kg body weight, since it was our first patient to be treated with prophylactic DLI and the patient was very young at the beginning of the treatment. In two adolescent patients (P#2 and P#6) we initially started with a higher dose of 1 × 10^6^ CD3^+^ cells/kg body weight, since this starting dose is more common among adult patients [16]. After both of these patients developed GvHD we treated all subsequent patients with a lower starting dose of 1 × 10^5^ CD3^+^ cells/kg body weight. The dose was successively increased each dose by 1 log if no signs of acute GvHD appeared. The intervals between consecutive DLI were approximately 6 weeks, as this was considered an optimal interval to guarantee a sufficient therapy density while allowing enough time to monitor possible toxicity. With this cautious dose escalation regimen and close clinical observation we applied up to a maximum dosage of 1 × 10^7^ CD3^+^ cells/kg body weight, although this dose is more commonly used in a therapeutic setting [12]. In case of signs of acute GvHD the administration of DLI was immediately discontinued. The regimen was the same for all patients, irrespective of donor type, age or disease burden. In patients with AML and ALCL a prophylactic treatment period of 2 years after allo‐SCT was pursued, in one patient with CML 5 posttransplant DLI were scheduled.

## 3 Results

### 3.1 Disease Outcome After Prophylactic DLI

**FIGURE 1:**
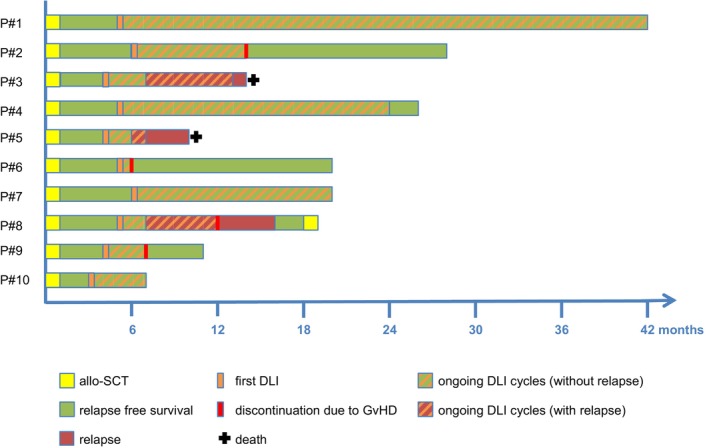
Clinical outcomes of patients receiving DLI.

In 10 patients, DLI was given prophylactically in the early posttransplantation period. Five patients had previously experienced relapse and four patients had been transplanted multiple times (range 2–3), leading to a very high overall‐risk profile of this study group. The median time from allo‐SCT to the first DLI administration was 4.7 months (range 3–6 months). Patients with AML and ALCL were treated until 2 years after allo‐SCT and received DLI approximately every 6 weeks. One patient with AML (P#1), who was treated after a third allo‐SCT, continued to receive DLI for approximately 3.5 years after the last allo‐SCT. In the latter patient, the interval between infusions has been extended to three months and, so far, no signs of toxicity have occurred. One patient with CML was scheduled to receive a total amount of 5 DLI after allo‐SCT every 6 weeks. In case of acute signs of toxicity (GvHD) the treatment was immediately discontinued. The average number of administered DLI was 7.1 (range 1–21). Three patients relapsed after DLI administration and subsequently received pre‐emptive DLI. One of those patients was re‐transplanted after another complete remission (CR) was achieved by polychemotherapy; the other two patients succumbed to their malignancy. The remaining seven patients are in CR and alive at follow‐up, resulting in an EFS of 70%. The median follow‐up after allo‐SCT was 19 months (range 7–42 months). OS was 80% and the median RFS was 17.2 months (range 5–42 months). Individual outcomes and survival are demonstrated in Figure 1.

### 3.2 GvHD After DLI

Two patients developed acute and two patients chronic GvHD after DLI. The affected organs were skin (*n* = 2), mucosa (*n* = 1) and lung (*n* = 1). Three cases (75% of all GvHD cases) presented as a mild GvHD and symptoms were controlled with local and systemic steroids as well as second line ruxolitinib before resolving completely. However, one patient developed a more extensive GvHD of the lung, meeting the diagnostic criteria of bronchiolitis obliterans syndrome (BOS). The patient was treated with a combination of fluticasone, azithromycin and montelukast in addition to systemic steroids, according to treatment recommendations for BOS published 2016 [17]. Hereunder the patient's symptoms and pulmonary function have continuously improved and are now resolved completely. All together high doses of donor lymphocytes were relatively well tolerated and did not lead to fatal complications (Table 2).

### 3.3 Individual Outcome After Third AML Relapse

P#1 was diagnosed with infant AML and relapsed 18 months after a first allo‐SCT (MSD). The patient was transplanted for a second time (haploidentical, MMFD) and showed incomplete donor chimerism 5 months after allo‐SCT. After two dosages of pre‐emptive DLI CR was restored but unfortunately the patient experienced a second overt hematological relapse 15 months after second allo‐SCT with poor response to therapeutic DLI. CR3 was achieved by intensive polychemotherapy but another relapse occurred after 17 months. The patient was transplanted a third time from a different donor (haploidentical, MMFD) and subsequently received prophylactic DLI, starting 5 months after the third allo‐SCT. Due to the extremely high risk of relapsing again and no adverse side effects, the patient continued to receive DLI for approximately 3.5 years after the last allo‐SCT. After two years of treatment the interval between infusions has been extended from 6 weeks to three months. The patient remains in CR and, so far, no signs of toxicity have occurred.

## 4 Discussion

DLI represent a well‐described immunotherapeutic approach to enhance a putative GvL‐effect after allo‐SCT, particularly in adult patients with hematological malignancies [9, 10]. However, its role in the pediatric setting remains insufficiently defined, with no standardized guidelines for indications, dosing regimens, or long‐term management. In this retrospective case series, we report our single‐center experience with DLI in children and adolescents treated for high‐risk hematological malignancies, focusing on clinical implementation and treatment‐related toxicities, particularly GvHD.

Our cohort, though small and heterogeneous in terms of diagnosis and treatment setting, provides valuable insights into the feasibility and safety of high‐dose and prolonged DLI schedules in pediatric patients. Notably, our approach included CD3^+^ T‐cell doses as high as 1 × 10^7^ cells/kg body weight. Furthermore, some patients received DLI over extended periods, including one patient who continues to receive DLI more than three years after the third allo‐SCT. These aspects distinguish our analysis from most previously published studies, which generally focus on adult populations [12] and shorter treatment timelines [14]. We hypothesize that prolonged DLI administration may be potentially beneficial in sustaining remission, especially in patients with persistent high relapse risk, but in this study, we merely describe an association of OS and RFS with DLI administration. The low toxicity observed during long‐term treatment supports the assumption that this treatment is feasible.

The most significant concern when administering DLI remains the induction of GvHD, which can offset any potential benefit by increasing transplant‐related morbidity and mortality [18]. In our cohort, two patients developed acute GvHD and two moderate chronic GvHD, affecting primarily the skin, mucosa, and lung. Importantly, no cases of severe acute or high grade GvHD were observed, even though 50% of our patients were transplanted from a haploidentical donor (MMFD). All patients were treated with standard immunosuppressive therapies, including systemic steroids and second line ruxolitinib, and no fatal complications occurred. The fact that the majority of our patients (*n* = 6) received bone marrow transplants may have contributed to the low rate and mild manifestation of GvHD in our cohort. Randomized trials have shown that using bone marrow as grafter source reduces the risk of developing GvHD compared to peripheral blood stem cells (PBSCs) [19]. These findings, though made in a limited cohort, are encouraging and might indicate that, even at higher T‐cell doses, DLI can be safely administered in pediatric patients. This also applies to a haplo‐identical setting, provided close monitoring and timely intervention are ensured. The dose‐escalation strategy and discontinuation of treatment upon early signs of GvHD likely contributed to the favorable safety profile observed in our cohort.

With respect to clinical efficacy, our cohort showed a notably good outcome considering the very high‐risk profile. Five of our patients had experienced relapses prior to the last allo‐SCT, two patients one and three patients even more, resulting in a very poor prognosis. Yet, seven of our patients remain in CR at a median follow‐up of 19 months.

When looking at the outcome of pediatric patients with AML, which represent the majority of our cohort (*n* = 8), the prognosis for relapsed patients is very poor. After a first relapse 5‐year survival is approximately 42% [20], after a second relapse it decreases to only 15% [21] and for patients with first relapse and high‐risk characteristics at initial diagnosis the OS is approximately 31% [20]. In our cohort, the OS was 50% for relapsed patients with AML, even though some of our patients had additional risk factors that generally deteriorate the outcome even more. Two patients had experienced multiple relapses and four patients had an unfavorable molecular risk profile. This might indicate that the administration of post allo‐SCT DLI could improve OS for pediatric patients with AML after relapse. But also the subgroup of AML patients that were transplanted in CR1 shows a better outcome than comparable cohorts. All of our AML patients (*n* = 4) that received prophylactic DLI after allo‐SCT in CR1 are alive and in CR at last follow‐up, resulting in an OS and EFS of 100%. General OS for pediatric patients with AML after allo‐SCT in CR1 is reported to be 60% [22]. Thus prophylactic DLI might also improve OS for pediatric patients with AML transplanted in CR1. However, it needs to be considered that this study only included a small number of cases and that it is not possible to draw statistically significant conclusions. The reference to pediatric AML outcomes can only be seen as a contextualization of our results.

In one individual patient (P#1) we also observed a long‐term remission‐free survival under prophylactic DLI after an insufficient response to preemptive and therapeutic DLI. This observation coincides with previously published data, suggesting the superiority of prophylactic over preemptive DLI with regard to long‐term survival [16, 23].

However, it has to be taken into consideration that some patients also received concomitant immunomodulatory medication that may have contributed to the favorable outcome. Additionally, only patients in a good general state of health with no acute posttransplant complications (GvHD, infection) were included in this study, leading to a possible selection bias.

Another limitation of our analysis is the heterogeneity of the cohort, in terms of underlying diseases (AML, CML, ALCL) as well as transplantation history (including second and third allo‐SCTs) and DLI administration regimen (various initial T‐cell doses). Furthermore, our observational analysis lacks a control group, and the sample size is too small to draw statistically significant conclusions. However, these limitations are inherent to the nature of rare pediatric diseases and reflect the need for randomized studies to define evidence‐based protocols for DLI in children.

In summary, our findings support the feasibility of high‐dose, extended prophylactic DLI in a small cohort of pediatric patients after allo‐SCT, even after use of haploidentical grafts. The observed GvHD rates and extent were controllable, and no patient suffered from severe complications. These results contribute to the growing body of evidence suggesting that DLI can be safely and effectively integrated into posttransplant care for selected pediatric patients. Nevertheless, interpretation of our case series is limited due to low patient numbers and patient heterogeneity. However, the results are very encouraging and underscore the urgent need for prospective studies and harmonized guidelines of DLI use in children and adolescents. Until such data are available, individualized treatment strategies based on risk stratification, close monitoring, and early recognition of toxicity will remain the cornerstone of safe and effective DLI application in pediatric hematology.

## Author Contributions

The study was designed by Denise Epple and Uwe Thiel. Data collection was performed by Katja Gall, Luisa Paschke, Angela Wawer, and Denise Epple. Hans Knabe manufactured the DLI. Denise Epple analyzed the data and wrote the first draft of the manuscript. Uwe Thiel, Irene von Luettichau, Hans‐Jochem Kolb, and Julia Hauer critically reviewed the manuscript for important intellectual content. All authors reviewed and approved the final manuscript.

## Funding

The authors declare financial support was received for the research, authorship, and/or publication of this article. This analysis has been facilitated due to generous funding to UT by the “Initiative Krebskranke Kinder e.V.” U.T. is also funded by the Deutsche Forschungsgemeinschaft (DFG, German Research Foundation)—project number 501830041, the “Zukunft Gesundheit e.V.,” and the Dr. Sepp und Hanne Sturm Memorial Foundation. U.T. is further funded by the Dr. Robert Pfleger‐Foundation and by the Wilhelm Sander‐Foundation (2018.072.1. and 2021.007.1.).

## Conflicts of Interest

The authors declare no conflicts of interest.

## Data Availability

The data that support the findings of this study are available from the corresponding author upon reasonable request.
